# A prospective algorithm for pre-operative risk stratification and individualized lymph node assessment in patients with endometrial intraepithelial neoplasia (EIN)^☆^

**DOI:** 10.1016/j.gore.2026.102177

**Published:** 2026-07-31

**Authors:** Pamela N. Peters, Lauren March, Georgia D. Smith, Elizabeth A. Bloom, Rebecca A. Previs, Angeles Alvarez Secord, Laura J. Havrilesky, Emma C. Rossi

**Affiliations:** a Sutter East Bay Medical Group, Berkeley, CA, United States of America; b Division of Gynecologic Oncology, Department of Obstetrics & Gynecology, Duke University, Durham, NC, United States of America; c Department of Obstetrics & Gynecology, Beth Israel Deaconess Medical Center, Boston, MA, United States of America; d Labcorp, Durham, NC, United States of America

**Keywords:** Endometrial intraepithelial neoplasia, Sentinel lymph node biopsy, Endometrial cancer, Surgical staging, Endometrial stripe, Pelvic ultrasound

## Abstract

**Objectives:**

The standard of care surgical management of endometrial intraepithelial neoplasia (EIN) is debated. Because EIN carries a 35–40% risk of underlying carcinoma, omission of lymph node sampling can result in incomplete staging. However, lymph node assessment for all patients results in overtreatment for those without cancer. We developed a preoperative risk-based algorithm to direct sentinel lymph node biopsy (SLNB) in EIN.

**Methods:**

Patients with EIN planning hysterectomy were treated prospectively according to our algorithm. Patients with an endometrial stripe (EMS) ≥ 15 mm by pre-operative ultrasound or “EIN-cannot rule out carcinoma” on pathology underwent indocyanine green (ICG) injection and SLNB. Patients with EMS < 15 mm underwent hysterectomy without SLNB.

**Results:**

From August 2022–November 2023, 50 patients with EIN underwent hysterectomy. 48% (24/50) had cancer on final pathology, and 16% (8/50) had high-risk cancers (defined as meeting high-intermediate risk criteria for adjuvant radiation, high grade histology, or stage II or higher). SLNB was performed in 92% (22/24) of patients with cancer, including 100% (8/8) with high-risk cancers. Surgical decision making based on the algorithm had 92% sensitivity for achieving surgical staging of cancer and 100% sensitivity for staging high-risk cancers. The negative predictive value of the algorithm (NPV) to correctly identify patients without cancer was 89%.

**Conclusions:**

A preoperative risk-based algorithm using EMS and pathology results has high sensitivity to identify and direct nodal assessment for patients with EIN at higher risk for endometrial cancer. The algorithm may allow for de-escalation and individualization of surgical treatment for EIN.

## Highlights

• An algorithm using endometrial stripe and pathology report has high sensitivity for detection of cancer on final pathology.
• This pre-operative decision-making algorithm can guide use of sentinel lymph node biopsy in EIN.
• Prospective implementation of this algorithm was associated with decreased operative time and frozen section use.

## 1 Introduction

Endometrial intraepithelial neoplasia (EIN) is a precursor to endometrial carcinoma, often conceptualized as carcinoma in situ of the endometrium. Up to 40% of patients diagnosed with EIN by endometrial sampling (endometrial biopsy or D&C) ultimately have invasive carcinoma on final hysterectomy specimen (Leitao Jr. et al., 2010). Most patients with pre-operative EIN who are upstaged to endometrial cancer at definitive surgery exhibit low-risk pathologic features, resulting in a low incidence of lymph node metastases. However, 5–10% have “high-risk” features where nodal assessment provides clinically relevant information for treatment planning (Costales et al., 2014; Matanes et al., 2022; Mueller et al., 2022; Sullivan et al., 2021). Patients with “low-risk” features are typically managed with observation without adjuvant therapy is reasonable even in the absence of complete staging, however “high-risk” features such as deep myometrial invasion, lymphovascular space invasion (LVSI), high grade histologies, or positive lymph nodes typically warrant adjuvant treatment with radiation and/or chemotherapy. The specific adjuvant treatment prescribed in these situations depends on whether the disease is node-positive or node-negative.

This creates a clinical challenge for referral strategy and surgical decision-making. The standard of care surgery for endometrial carcinoma is hysterectomy with lymph node assessment, either by sentinel lymph node (SLN) mapping and biopsy or complete lymphadenectomy (NCCN, 2024; Rossi et al., 2017). Sentinel lymph node biopsy (SLNB) provides prognostic information to guide treatment and is associated with less morbidity than complete lymphadenectomy (Leitao Jr. et al., 2020). SLN mapping requires injection of a tracer (typically indocyanine green [ICG]) into the cervix prior to the disruption of lymphatic channels by hysterectomy; this requires a decision to perform SLNB to be made prior to hysterectomy. The American College of Obstetrics and Gynecology (ACOG) and Society of Gynecologic Oncology (SGO) guidelines on EIN recommend hysterectomy but do not address SLNB. (Parkash et al., 2015)

Despite the absence of clinical guidelines to explain a change in practice, the rate of SLNB in patients with EIN has significantly increased over time, from 0.6% of EIN cases in 2012 to 26.1% in 2020 (Dioun et al., 2021; Levin et al., 2024). When considering SLNB for EIN, surgeons must balance the probability of overtreatment by performing SLNB for all patients with EIN, versus the probability of incomplete staging for patients with invasive carcinomas. Additionally, only gynecologic oncologists perform SLNB, creating uncertainty about whether referral to gynecologic oncology is warranted for EIN. Without preoperative risk stratification, some surgeons utilize intraoperative frozen section to rule out high-risk cancers in patients with EIN, however this approach is not ideal. Frozen section has poor inter-rater reliability, increases cost and time in the operating room, and requires use of the hysterectomy specimen, thus losing the opportunity to perform SLNB and requiring complete lymphadenectomy, which may include pelvic and para-aortic dissection, if nodal sampling is indicated (Case et al., 2006; Frumovitz et al., 2004; Lim et al., 2018). For patients with EIN, there are no prospective data available to pre-operatively stratify those at the highest risk of invasive cancer to triage the need for SLNB.

Pre-operative stratification of patients with EIN to determine risk of cancer would be clinically useful in triaging referral to gynecologic oncology and need for sentinel lymph node biopsy.

We created an algorithm to prospectively risk-stratify patients prior to surgical management of EIN. The algorithm was used to assign patients to hysterectomy alone (no ICG injection) or SLNB and hysterectomy (ICG injection). We aimed to evaluate the sensitivity, specificity, and positive/negative predictive value to detect cancer and high-risk cancer using this strategy.

## 2 Methods

We designed a prospective cohort study at a single institution with seven gynecologic oncologists. This study was reviewed by the Institutional Review Board (IRB) (IRB #Pro00110354) and determined to be exempt as a quality improvement project. It was the consensus of our group that the algorithm met standard of care, and the primary goal of this project was improvement in quality of surgical care for patients with EIN.

The authors reviewed the evidence to identify factors associated with increased risk of invasive carcinoma on final pathology in patients with EIN. We identified several retrospective studies that demonstrated increased endometrial stripe thickness (Vetter et al., 2020; Abt et al., 2022), inability to “rule out” carcinoma on pathology (Touhami et al., 2018), and *ARID1A* mutations (Yen et al., 2018) as predictors of invasive carcinoma. We created an algorithm incorporating endometrial stripe thickness and pathology report language as these pieces of clinical information are frequently already available when surgical decisions are made. We selected an endometrial stripe thickness of 15 mm as a threshold for surgical decision-making based on published retrospective data suggesting endometrial stripe ≥15 mm was associated with increased rates of endometrial cancer with high-risk features (Abt et al., 2022). Pathology report language that suggests inability to rule out carcinoma in addition to diagnosis of EIN was associated with increased risk of lymph node metastases (Touhami et al., 2018). Both associations with higher risk disease were identified retrospectively, and we aimed to evaluate these prospectively.

In our algorithm, patients “EIN-cannot rule out carcinoma” or other similar language on pathology, or with an endometrial stripe (EMS) ≥ 15 mm by pre-operative ultrasound, underwent indocyanine green (ICG) injection and SLNB (cohort 1). For patients with EMS < 15 mm, ICG injection was not performed, and decision-making regarding nodal assessment was at surgeon discretion (cohort 2). If pelvic ultrasound was not available at time of consultation, it was ordered and obtained prior to surgery unless pathologic criteria alone were sufficient for assignment to cohort 2. Frozen section was performed at surgeon discretion and could be utilized if preoperative ultrasound was unavailable, or as a complementary intraoperative assessment, including to guide further nodal assessment in cases of failed sentinel lymph node mapping. We hypothesized that the algorithm would have high sensitivity for detection of high-risk cancer.

Prior to algorithm implementation, we performed a retrospective analysis of select patients who underwent surgery for EIN at our institution from January 2016–December 2021. This database included subjects with a diagnosis of EIN or endometrial cancer during this time frame who also had available tissue within our BioRepository and Precision Pathology Center (BRPC) and represented a sample of patients with EIN rather than a comprehensive cohort. Within this database we identified 77 patients with a pre-operative diagnosis of EIN. 23 patients (30%) had cancer on final pathology, and 5 patients (6.5%) had high-risk cancer. The baseline rate of lymph node assessment in patients with high-risk cancer was 60% (3/5 patients), and 9% of patients without cancer underwent lymph node assessment. Theoretical use of the proposed algorithm on the retrospective cohort, 100% of patients with cancer would have been appropriately selected for surgical staging, and 21% of patients with a final diagnosis of EIN would have undergone surgical staging. These data as well as the retrospective data in the literature were presented at gynecologic oncology division meeting to create a consensus on the planned algorithm prior to implementation. We created a plan for interim analyses and detailed chart review at intervals of 10 patients to evaluate for safety signals, such as under staging of high-risk cancers. Our goal was to capture 80% of patients with high-risk cancer for surgical staging (compared to baseline rate of 60% at our institution).

**Fig. 1:**
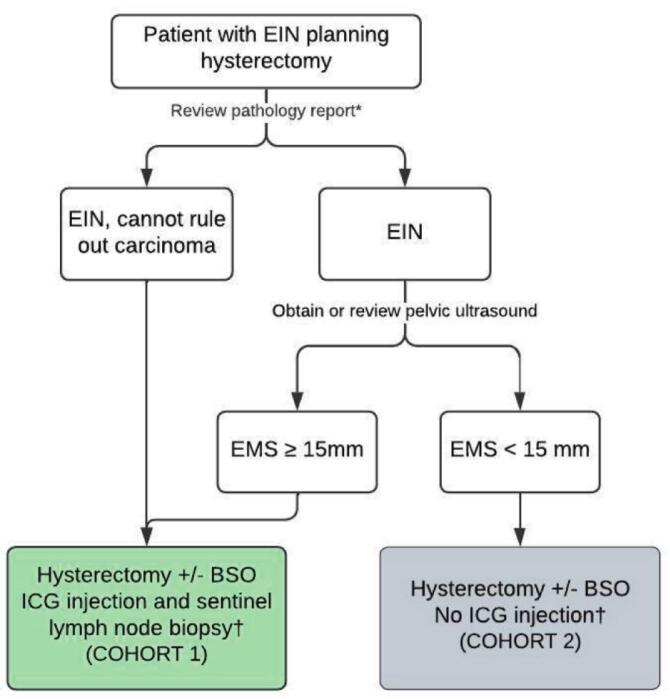
**Pre-operative risk stratification algorithm of patients with EIN.** Patients who were planning hysterectomy were eligible for inclusion in risk-stratification algorithm. The pathology report was reviewed and if it included language about inability to rule out carcinoma or bordering on carcinoma, patients were selected for cohort 1. If pathology report described EIN, pelvic ultrasound was reviewed if available within the prior 6 months, or obtained if not available. If EMS was greater than or equal to 15 mm, patients were selected for cohort 1. If EMS was less than 15 mm, patients were selected for cohort 2. Treatment for cohort 1 included hysterectomy +/− BSO with ICG injection and sentinel lymph node mapping and biopsy. Cohort 2 included hysterectomy +/− BSO without ICG injection. *For pathology obtained outside our institution, internal pathology review was recommended but not required. ^†^Intra-operative frozen section was utilized at the discretion of the surgeon, and was recommended for patients with EIN who did not have pelvic ultrasounds performed pre-operatively. EIN = endometrial intraepithelial neoplasia; BSO = bilateral salpingo-oophorectomy; EMS = endometrial stripe; ICG = indocyanine green.

Patients with a pre-operative diagnosis of EIN, atypical endometrial hyperplasia, or complex endometrial hyperplasia undergoing hysterectomy were treated according to the algorithm from August 2022–November 2023 according to the algorithm (Fig. 1). Internal pathology review was recommended for outside pathology specimens as part of standard institutional practice. We collected data regarding demographic and clinical variables, route and type of surgery, utilization of frozen section, operative time, final pathology, and adjuvant treatments. The performance characteristics of the algorithm were evaluated by calculation of the sensitivity and specificity for detection of cancer and “high-risk” cancer, as well as positive and negative predictive values. “High-risk” cancer was defined as meeting Gynecologic Oncology Group (GOG) 99 and/or Post-Operative Radiation Therapy for Endometrial Carcinoma (PORTEC) high-intermediate risk criteria, high grade histology (grade 3, serous, clear cell, or undifferentiated), or FIGO 2009 stage II or higher (Creutzberg et al., 2000; Keys et al., 2004; Nout et al., 2010).

We defined sensitivity as the percentage of cancers surgically staged out of all patients with cancer. Sensitivity for high-risk cancer was defined as percentage of high-risk cancers surgically staged out of all patients with high-risk cancers. Positive predictive value was defined as the percentage of patients who underwent ICG injection and SLNB (cohort 1) who were ultimately diagnosed with cancer. Negative predictive value was defined as the percentage of patients who underwent hysterectomy alone (cohort 2) who were not diagnosed with cancer (EIN or less).

## 3 Results

Fifty consecutive patients undergoing hysterectomy for EIN from August 2022–November 2023 made up the study population. Of these patients, 32 received ICG injection (cohort 1) and 18 patients did not (cohort 2). ICG injection was performed in concordance with the algorithm in 86% (43/50) of cases; algorithm concordance was similar between the two cohorts (84% [27/32] in cohort 1, and 89% [16/18] in cohort 2). The median age was 60.5 years (IQR 32.3, 67); median BMI 39.0 kg/m^2^ (IQR 30.3, 42.2) and was similar between the two cohorts (Table 1). The study included 16 (32%) patients who identified as Black and 34 (68%) patients who identified as White. All (100%) patients in this study were non-Hispanic. Central pathology review at our institution to confirm the pre-operative diagnosis was performed in 70% of cases. Pelvic ultrasound was already available at time of initial consult in 80% of patients (40/50) and was obtained prior to surgery in 96% of patients (48/50). Median EMS differed between the two groups with median EMS of 15 mm (IQR 9.5, 17.5) in cohort 1 and 7 mm (IQR 5.5, 9.5) in cohort 2. Route of surgery was 54% (27/50) robotic, 44% (22/50) laparoscopic and 2% (1/50) open, with 38% (19/50) of cases utilizing frozen section. Route of surgery and frozen section utilization was similar between cohorts. Median operative time was 150 min in the entire cohort (IQR 120, 168), with a median time of 155 min (IQR 129, 170) in cohort 1 and 135 min (IQR 113, 158) in cohort 2 (Table 1).

**Table 1:** **Clinicopathologic characteristics of patients with EIN undergoing hysterectomy.** Patients were pre-operatively risk stratified into cohort 1 or cohort 2. Both cohorts underwent hysterectomy +/− bilateral salpingo-oophorectomy. Patients in cohort 1 received ICG injection and sentinel lymph node mapping and biopsy, and patients in cohort 2 did not receive ICG injection.

	Cohort 1 *ICG injected* (*n* = 32)	Cohort 2 *No ICG injected* (*n* = 18)	Total (*n* = 50)
**Age** (median, IQR**)**	60.5 (55.8, 67.5)	59 (52.0, 66.8)	**60.5 (52.3, 67)**
**Race** (n, %)	Black: 11 (34%) White: 21 (66%)	Black: 5 (28%) White: 13 (72%)	**16 (32%)** **34 (68%)**
**BMI** (median, IQR)	39.7 (34.2, 42.7)	36.5 (28.3, 41.6)	**39.0 (30.3, 42.2)**
**Internal pathology review** (n, %)	21 (66%)	14 (78%)	**35 (70%)**
**Pelvic ultrasound available at initial consult** (n, %)	27 (84%)	13 (72%)	**40 (80%)**
**EMS thickness in mm** (median, IQR)	15 (9.5, 17.5)	7 (5.5, 9.5)	**12 (7, 16)**
**ICG use concordant with algorithm**⁎ (n, %)	27 (84%)	16 (89%)	**43 (86%)**
**Intraoperative frozen section utilization** (n, %)	12 (38%)	7 (39%)	**19 (38%)**
**Route of surgery** (n, %)	MIS Robotic:17 (53%) MIS Laparoscopic: 14 (44%) Open: 1 (3%)	MIS Robotic:10 (56%) MIS Laparoscopic: 8 (44%) Open: 0 (0%)	**27 (54%)** **22 (44%)** **1 (2%)**
**Operative time** (median, IQR)	155 (129, 170)	135 (113, 158)	**150 (120, 168)**
**Final pathology** (n, %)	Cancer: 22 (69%) EIN or less: 10 (31%)	Cancer: 2 (11%) EIN or less: 16 (89%)	**24 (48%)** **26 (52%)**
**High-risk**⁎⁎**cancer on final pathology** (n, %)	8 (25%)	0 (0%)	**8 (16%)**
**Positive lymph nodes** (n, %)	0 (0%)	0 (0%)	**0 (0%)**

ICG = indocyanine green; IQR = interquartile range; BMI = body mass index; EMS = endometrial stripe; MIS = minimally invasive surgery; EIN = endometrial intraepithelial neoplasia.

⁎ Concordance as defined as ICG injection for patients assigned to cohort 1 and omission of ICG injection for patients assigned to cohort 2.

⁎⁎ High-risk cancer was defined as meeting GOG 99 and/or PORTEC high-intermediate risk criteria, or high-grade histology (grade 3, serous, clear cell, or undifferentiated), or stage II or higher.

In cohort 1 (ICG injection), 69% (22/32) of patients had invasive cancer on final pathology, compared to 11% (2/18) in cohort 2 (no ICG injection). There were 8 patients with high-risk cancers on final pathology. All 8 patients were in cohort 1 (ICG injection), and patients with high-risk cancers represented 25% of the patients in cohort 1 and 16% of the entire cohort. None of the patients had positive lymph nodes in this series (Table 1).

The algorithm was determined to have a 92% sensitivity (*n* = 22/24) and 63% specificity (*n* = 17/27) for detection of cancer in patients with pre-operative diagnoses of EIN. A positive algorithm had a 69% positive predictive value for cancer. The algorithm had 100% sensitivity (*n* = 8/8) for detection of high-risk cancer and a 30% positive predictive value (n = 8/27) for detection of high-risk cancer. A “negative algorithm”, defined as being selected for cohort 2, had an 89% negative predictive value for absence of cancer (*n* = 17/19), and 100% negative predictive value for lack of high-risk cancer (*n* = 24/24) (Table 2).

**Table 2:** Sensitivity and specificity for cancer and high-risk* cancer. (A) A “positive algorithm” defined as being selected for cohort 1 and ICG injection with sentinel lymph node biopsy, had a 92% sensitivity and 63% specificity for detection of cancer with a pre-operative diagnosis of EIN. The positive predictive value of a “positive algorithm” for cancer was 69%, and the negative predictive value of a “negative algorithm” for lack of cancer (EIN or benign) was 89%. (B) A “positive algorithm” had a 100% sensitivity and 56% specificity for detection of high-risk* cancer. The positive predictive value of a “positive algorithm” for high-risk cancer was 30%, and the negative predictive value of a “negative algorithm” for lack of high-risk cancer (low risk cancer, EIN, or benign) was 100%.

A		
	Cancer on final pathology	EIN or benign on final pathology
**“Positive algorithm” =** **ICG injection**	22	10
**“Negative algorithm” =** **No ICG injection**	2	17

B		
	High-risk⁎ cancer on final pathology	EIN or benign on final pathology
**“Positive algorithm” =** **ICG injection**	8	19
**“Negative algorithm” =** **No ICG injection**	0	24

EIN = endometrial intraepithelial neoplasia; ICG = indocyanine green.

⁎ High-risk cancer was defined as meeting GOG 99 and/or PORTEC high-intermediate risk criteria, or high-grade histology (grade 3, serous, clear cell, or undifferentiated), or stage II or higher.

Of the 32 patients in cohort 1, 12 patients (37.5%) ruled in by pathology report language, 8 (25%) by EMS, 8 (25%) by both pathology report language and EMS, and 4 (12.5%) did not rule in by either criterion (non-compliance with the algorithm). Of the four patients who received ICG injection who should not have by strict compliance with the algorithm, 3 had final diagnoses of EIN and 1 had a diagnosis of cancer with low-risk features.

**Fig. 2:**
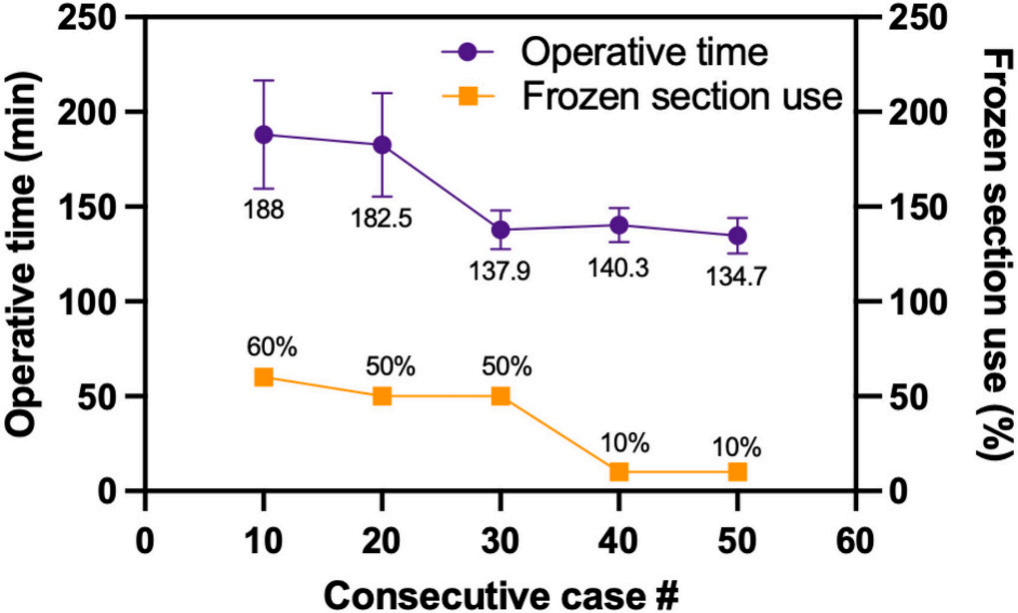
**Trends in operative time and frozen section utilization.** Operative time and frozen section utilization were tracked for 50 consecutive cases after implementation of the pre-operative decision-making algorithm. Mean operative time, in increments of 10 consecutive cases, was decreased over time from mean of 188 min to 134.7 min for cases 40–50. Each data point represents the mean for the 10 prior consecutive cases with error bar representing standard error of the mean. Frozen section utilization decreased over time from 60% of the first 10 cases to 10% in cases 40–50.

As the algorithm was implemented, we noted trends towards decreased use of frozen section, from 6/10 of the first ten cases to 1/10 of the last ten cases. There were also trends of decreasing operative time, from a mean of 188 min in the first ten cases to 135 min in the last ten cases (Fig. 2).

### 3.1 Subset characterization of patients with high-risk endometrial cancers

The clinicopathologic characteristics of the eight patients with high-risk cancers are described in Table 3. Of patients with high-risk cancers, 50% were Black and 50% were White. EIN was initially diagnosed by EMB in 75% (6/8), and D&C or HSC/D&C in 25% (2/8). 50% (4/8) of patients were selected for cohort 1 based on pathology report language, 37.5% (3/8) by EMS and 12.5% (1/8) by both pathology and EMS. One patient had a stage IIIA endometrioid carcinoma with microsatellite instability (MSI-H). Three patients had stage IA high grade serous carcinomas, characterized by abnormal p53 protein expression, a surrogate of *TP53* mutations. Four patients had stage I endometrioid carcinomas with normal p53 protein expression consistent with an intact *TP53* gene (Table 3). Of the 8 patients with high-risk cancers, all were recommended to receive adjuvant treatment after surgery. Patients were treated with vaginal brachytherapy (VBT) alone (*n* = 3), carboplatin and paclitaxel chemotherapy and VBT (*n* = 2), carboplatin, paclitaxel, pembrolizumab and with pembrolizumab maintenance therapy (*n* = 1). Two patients declined adjuvant therapy.

**Table 3:** **Detailed clinicopathologic features of patients with high-risk endometrial cancers.** BMI = body mass index; EMS = endometrial stripe; US = ultrasound; HSC = hysteroscopy; D&C = dilation and curettage; EMB = endometrial biopsy; TLH = total laparoscopic hysterectomy; BSO = bilateral salpingo-oophorectomy; SLND = sentinel lymph node biopsy; ra = robotic-assisted; LVSI = lymphovascular space invasion; myo = myometrial; MSS = microsatellite stable; MSI-H = microsatellite instability high; VBT = vaginal brachytherapy.

Age (years)	Race	BMI (kg/m^2^)	EMS on US	Sampling method	Pre-operative pathology	Internal pathology review	Surgery	Frozen section	Final Pathology	Molecular features	Adjuvant treatment
70	White	30	36 mm	D&C	EIN at least, suspicious for adenocarcinoma	Yes	TLH, BSO, bilateral SLNB, washings	Yes Risk factors present	Stage IB endometrioid adenocarcinoma Grade 1 3.8 cm tumor 75% myo invasion No LVSI Negative SLNs	p53 wild type MSS	VBT (3000 cGy)
81	Black	31	25 mm	EMB	EIN	No	TLH, BSO, bilateral SLNB	No	Stage IA serous carcinoma confined to a polyp Grade 3 No myo invasion No LVSI Negative SLNs	p53 mutated MSS Her2neu neg	None (Recommended chemotherapy and VBT)
61	White	41	12 mm	HSC/ D&C	EIN at least, suspicious for adenocarcinoma	No	ra-TLH, BSO, bilateral SLNB, washings	No	Stage IB endometrioid adenocarcinoma Grade 1 4.5 cm tumor 85% myo invasion No LVSI Negative SLNs	p53 wild type MSS	VBT (3000 cGy)
58	White	40	5 mm	EMB	EIN at least, suspicious for adenocarcinoma	Yes	TLH, BSO, bilateral SLNB, washings	Yes No risk factors present	Stage IA endometrioid adenocarcinoma Grade 2 1.7 cm tumor 16% my invasion Focal LVSI Negative SLNs	p53 wild type MSS	VBT (3000 cGy)
76	Black	36	34 mm	EMB	EIN	No	ra-TLH, BSO, unilateral SLNB	Yes No risk factors present	Stage IA endometrioid adenocarcinoma Grade 2 6.6 cm tumor 40% myo invasion No LVSI Negative SLNs	p53 wild type MSS	None (Recommended VBT)
71	Black	42	17 mm	EMB	EIN	Yes	ra-TLH, BSO, bilateral SLNB	No	Stage IA serous carcinoma of the endometrium Grade 3 1 cm tumor confined to a polyp No myo invasion No LVSI Negative SLNs	p53 mutated MSS Her2neu pos	Chemotherapy (6 cycles) and VBT (3000 cGy)
69	Black	41	“Normal”	EMB	EIN at least, suspicious for adenocarcinoma	Yes	ra-TLH, BSO, bilateral SLNB	No	Stage IA serous carcinoma of the endometrium Grade 3 4.4 cm tumor arising in a polyp No myo invasion No LVSI Negative SLNs	p53 mutated MSS Her2neu neg	Chemotherapy (6 cycles) and VBT (3000 cGy)
71	White	24	NA, not performed	EMB	EIN at least, suspicious for adenocarcinoma	No	ra-TLH, BSO, bilateral SLNB, washings	No	Stage IIIA endometrioid adenocarcinoma Grade 2 6.6 cm 100% myo invasion with serosal involvement Extensive LVSI Negative SLNs	p53 wild type MSI-H;	Chemotherapy (6 cycles) and immunotherapy

## 4 Discussion

In this study of patients with pre-operative diagnosis of EIN, an algorithm incorporating pre-operative pathology and pelvic ultrasound had a 100% negative predictive value for ruling out endometrial cancer with high-risk features. Utilizing readily clinical available information, the algorithm was associated with a low rate of unnecessary staging procedures in patients without invasive cancer, and with decreasing operative time and use of frozen section throughout the study period. This approach provides a clear pre-operative framework that balances the competing risks of overtreatment of EIN versus under staging of high-risk cancers.

There are no formal guidelines regarding extent of surgical staging for EIN (NCCN, 2024; *Obstet. Gynecol.*, 2023). Current strategies include no nodal assessment, routine SLNB, or intra-operative decision making based on frozen section. Routine SLNB for EIN has been shown to be safe (Mueller et al., 2022) and may be cost effective (Dioun et al., 2021; Lim et al., 2018; Walker et al., 2025); however, its universal use would result in unnecessary staging for nearly half of all patients with EIN, who ultimately do not have invasive carcinoma. Additionally, universal SLNB in EIN can create additional burden for patients, particularly in low-resource settings, by introducing delays in care, travel cost and lose wages that result from referral to gynecologic oncology (Desjardins et al., 2023). With collection of more data to confirm our findings, the current study suggests that omission of nodal assessment – and potentially referral to gynecologic oncology— is likely appropriate when the endometrial stripe is less than 15 mm and there are no pathologic features concerning for carcinoma. However, validation of this approach across referring and receiving specialty practices is needed.

Predictive factors for occult invasive cancer in the setting of preoperative EIN have been identified in retrospective studies and include endometrial thickness, pathology report language and molecular markers such as *ARID1A* (Vetter et al., 2020; Abt et al., 2022; Touhami et al., 2018; Yen et al., 2018). We selected pragmatic, affordable clinical variables – ultrasound findings and routine histopathology – that are generally available at the time of diagnosis. Incorporation of additional clinical and molecular predictors including age, metabolic factors, race and immunohistochemistry stains to *ARID1A*, p53, and mismatch repair proteins may further refine risk-stratification (Puechl et al., 2021). However, this initial study was designed to evaluate a feasible and broadly applicable approach to minimize unnecessary nodal assessment while reducing the risk of incomplete staging.

Consistent with prior studies, nodal metastases were rate in this population, with no positive lymph nodes identified (Leitao Jr. et al., 2010; Costales et al., 2014; Matanes et al., 2022; Mueller et al., 2022). However, the value of nodal assessment extends beyond the identification of metastases. Confirmation of negative nodal status may also inform adjuvant treatment decisions. In our cohort, 16% of patients with preoperative EIN were ultimately diagnosed with invasive cancer with “high-risk” features. Patients with uterine-confined disease and high-risk features such as high-grade histology, lympho-vascular space invasion and deep myometrial invasion, may be recommended to receive adjuvant radiation therapy to reduce risk of local recurrence (Creutzberg et al., 2000; Keys et al., 2004). In the absence of nodal metastases, vaginal brachytherapy alone has been shown to be adequate for many of these patients (Nout et al., 2010). Although absence of surgical nodal assessment does not itself mandate external beam radiation therapy (EBRT), observational data suggest that clinicians are more likely to prescribe pelvic EBRT when high-risk features are identified and nodal status is unknown (Sharma et al., 2011). External beam radiation confers more toxicity, particularly chronic gastrointestinal and urologic complaints, compared with vaginal brachytherapy (Nout et al., 2010). Therefore, in some patients with high-risk uterine-confined disease, omission of lymph node assessment may contribute to use of more extensive adjuvant radiation approach than would otherwise be selected, or prompt consideration of a second procedure for surgical staging (NCCN, 2024). These considerations underscore the value of identifying patients at risk for occult carcinoma with “high-risk” features before proceeding with hysterectomy alone.

Frozen section utilization and operative time decreased over the study period, suggesting a more efficient and streamlined operative strategy. Frozen section had been routinely utilized by several surgeons prior to implementation of the algorithm, and its declining use over the study period likely reflects increasing surgeon familiarity and confidence with the algorithm as experience accumulated. This approach may have implications for the cost and efficiency of EIN management through reduced reliance on frozen section, more predictable operative planning, and more selective subspeciality referral.

Strengths of this study include its prospective design and implementation of a standardized surgical approach across a division of gynecologic oncologists. Conducting the study at a single institution facilitated consistency but may limit generalizability. Although the sample size was small, it included a diverse population of patients, with 32% Black patients, an important consideration given the performance of endometrial thickness as a predictive measure may differ across racial groups (Doll et al., 2021). However, limited representation of other racial and ethnic groups limits generalizability.

Pre-operative review of endometrial biopsy samples by specialist gynecologic pathologists at a tertiary referral center occurred in 70% of cases in this study, limiting the applicability to less specialized settings. However, the inclusion of patients with diagnoses of complex atypical hyperplasia and atypical hyperplasia, often rendered at referring institutions, reflects the real-world heterogeneity of pathologic terminology. Although these diagnoses are not strictly synonymous with EIN, they represent substantially overlapping disease categories and have frequently been considered together the literature (Emons et al., 2015).

Additional limitations include the small sample size, which precluded evaluation of patients with nodal metastases, and our inability to evaluate whether preoperative sampling method, including hysteroscopy and D&C versus pipelle biopsy, impacted algorithm performance. Finally, the absence of baseline data for operative time and frozen section use before study implementation prevents us from claiming conclusively that our algorithm decreases operative time.

Further refinement and external validation of this algorithm is necessary. It remains important to understand how menopausal status impacts the threshold of endometrial thickness, and whether incorporation of routine molecular subtyping, such as those defined in the ProMisE algorithm, can improve risk stratification (Kommoss et al., 2018). All four ProMisE molecular categories have been identified in EIN (Puechl et al., 2021), although the clinical utility of applying this framework to EIN remains uncertain. Incorporation of prognostic molecular features, such as abnormal p53 expression, may further improve identification of patients likely to have high-risk carcinoma. Further studies should also evaluate comparative cost-effectiveness of this strategy compared to others in diverse practice settings. In the absence of consensus guidelines regarding nodal assessment for EIN, an approach incorporating endometrial thickness and pathologic features concerning for carcinoma may provide a pragmatic strategy to selectively identify patients most likely to benefit from surgical staging.

## CRediT authorship contribution statement

**Pamela N. Peters:** Writing – review & editing, Writing – original draft, Visualization, Methodology, Investigation, Formal analysis, Data curation, Conceptualization. **Lauren March:** Writing – review & editing, Project administration, Formal analysis, Data curation. **Georgia D. Smith:** Writing – review & editing, Investigation, Data curation. **Elizabeth A. Bloom:** Writing – review & editing, Methodology. **Rebecca A. Previs:** Writing – review & editing, Methodology, Formal analysis. **Angeles Alvarez Secord:** Writing – review & editing, Supervision, Methodology. **Laura J. Havrilesky:** Writing – review & editing, Supervision, Methodology. **Emma C. Rossi:** Writing – review & editing, Supervision, Methodology, Conceptualization.

## Ethics approval

This study was reviewed by the Duke University Institutional Review Board (IRB #Pro00110354) and determined to be exempt.

## Funding

This research did not receive any specific grant from funding agencies in the public, commercial, or not-for-profit sectors.

## Declaration of competing interest

Dr. Rebecca Previs has no direct disclosures of interest related to this topic. She does report she is employed by and owns stock in Labcorp. Dr. Alvarez Secord has no direct disclosures of interest related to this topic. She does report that her institution has received clinical trial grant funding from Abbvie, Aravive, Astra Zeneca, Clovis, Eisai, Ellipses Pharma, Genmab, GSK, Immunogen, Karyopharm, Merck, Mersana, Myriad, OncoQuest/Canaria Bio, Roche/Genentech, Seagen Inc., TORL Biotherapeutics, VBL Therapeutics, and Zentalis. She has participated in Advisory Board for AbbVie/Immunogen, GSK and Histosonics and uncompensated Advisory Boards for AstraZeneca, Gilead, Imvax, and Oncoquest/CanariaBio; and on Clinical Trial Steering Committees (uncompensated) for the Oval Trial (VBL Therapeutics), FLORA-5 and FLORA-4 trials (Oncoquest); received stock options in Amgen and Johnson & Johnson and royalites from UpToDate; participation on the AAOGF Board of Trustees, SGO Board of Directors, FWC Board of Directors, and GOG Foundation Board of Directors; payment for lectures for Research to Practice, Clinical Care Options. Dr. Emma Rossi has no direct disclosures of interest related to this topic. She has consulted for Medtronic and Intuitive.
